# Optimizing DNA recovery and forensic typing of degraded blood and dental remains using a specialized extraction method, comprehensive qPCR sample characterization, and massively parallel sequencing

**DOI:** 10.1007/s00414-019-02124-y

**Published:** 2019-08-14

**Authors:** Patricio Carrasco, Carolina Inostroza, Meghan Didier, Marianela Godoy, Cydne L. Holt, Jonathan Tabak, Andrew Loftus

**Affiliations:** 1 Universidad de los Andes, Mons. Álvaro del Portillo 12.455, Las Condes, Santiago Chile; 2 Verogen, Inc., 11111 Flintkote Avenue, San Diego, CA 92121 USA; 3 InnoGenomics Technologies, LLC, 1441 Canal Street, New Orleans, LA 70112 USA

**Keywords:** Degraded DNA, Single-Nucleotide Polymorphism (SNP), Massively Parallel Sequencing (MPS), ForenSeq, InnoQuant HY, Dental Forensic Kit (DFK)

## Abstract

Human dental remains encountered in criminal casework evidence, missing person cases, or mass disaster tragedies provide a valuable sample source for DNA typing when suitable soft tissue is unavailable. Using traditional methods, teeth samples can be challenging to process, resulting in low-quantity and/or quality nuclear DNA and insufficient profiles for comparisons. This study examines the performance of a three-part nuclear DNA analysis workflow for teeth samples based on (1) improved dental tissue recovery using the Dental Forensic Kit (DFK^MR^) (Universidad de los Andes) and DNA extraction with QuickExtract™ FFPE DNA Extraction Kit (Lucigen®), (2) quantification with InnoQuant® HY (InnoGenomics Technologies) for sensitive assessment of total human and male DNA quantity/quality, and (3) massively parallel sequencing for simultaneous genotyping of 231 short tandem repeat (STR) and single-nucleotide polymorphism (SNP) markers with the ForenSeq® DNA Signature Prep Kit (Verogen, Inc.). Initial evaluation of artificially degraded blood samples (*n* = 10) achieved highly sensitive and informative quantification results with InnoQuant® HY, enabling successful first pass genotyping with the MiSeq FGx® System. Twenty-three STR alleles (out of 85) and 70 identity informative SNP loci (out of 94) were recovered from two pg total *long target DNA input* (0.86 ng total *short target input*) and an InnoQuant degradation index (DI) of 460 (severely degraded). The three-part workflow was subsequently applied to teeth samples (dental pulp, root cement tissues; *n* = 13) with postmortem intervals (PMI) of the teeth ranging from 8 days to approximately 6 months. Informative SNP and STR DNA profiles were obtained, to include 78 STR alleles and 85 identity informative SNP loci typed (of 94 total SNP targets) in a 1 month, four-day PMI root cement sample with one pg total *long target DNA input* and a DI of 76. These data indicate successful performance of the proposed workflow from degraded DNA from teeth samples.

**Electronic supplementary material:**

The online version of this article (10.1007/s00414-019-02124-y) contains supplementary material, which is available to authorized users.

## Introduction

Forensic DNA analysis often comprises challenging samples caused by partial DNA degradation, presence of PCR inhibitors, low DNA quantities, and/or mixtures of multiple contributors. These variables can require iterative rounds of time-consuming testing to generate sufficient data for comparisons and identifications. Dental remains (i.e.*,* teeth samples) are commonly subjected to these variables and can be the sole DNA source when viable nuclear DNA are not available due to soft tissue decomposition [1, 2]. Improved upstream sample collection and cellular recovery, in combination with a highly informative quantification assay and deep, targeted massively parallel sequencing (MPS) analysis collectively maximize the genetic information potential from dental remains. Application of an optimized workflow that combines extraction, quantification, and MPS protocols was evaluated to assess performance on forensic dental samples.

The Dental Forensic Kit (DFK^MR^) was specifically designed to optimize dental tissue retrieval in routine forensic nuclear DNA and/or mitochondrial DNA genotyping and forensic histopathology applications [1–3]. By conducting dental tissue preparation (dental pulp, root cement) in physiological conditions, the DFK method maximizes DNA retrieval and thus genetic information potential from challenging samples. Attributes of the DFK method that make it a feasible alternative to other methodologies for hard tissues [4, 5] include faster processing time to obtain two distinct tissue samples (dental pulp, root cement), decreased contamination (grinding not required), and tooth preservation for further re-extraction or additional testing, if needed. Additionally, the method’s non-destructive aspect has cultural and/or religious implications in that what may be the sole remains for burial can be handled in the most sensitive manner. After tissue retrieval with DFK, the QuickExtract™ FFPE DNA Extraction Kit (Lucigen®) was used to effectively extract nuclear DNA [6, 7].

Quantity and quality of total human and total male DNA can be assessed using the InnoQuant® HY Kit (InnoGenomics Technologies) [8, 9]. Based on real-time qPCR quantification of high copy number Alu and SVA retrotransposable elements (> 1000 copies per genome), this method assesses autosomal targets of two lengths (short and long) to enhance sensitivity and reproducibility of DNA quantitation and degradation detection in forensic samples [8, 9]. Quality information includes detection of polymerase chain reaction (PCR) inhibitors and determination of a degradation index (DI), which correlates strongly to amplification and genotyping success [8, 9]. The InnoQuant HY method was integrated as the second portion of the three-part workflow.

The last portion of the comprehensive workflow is massively parallel sequencing (MPS), also known as next-generation sequencing (NGS), which was assessed for ability to relieve limitations of amplicon length-based STR allele calling after capillary electrophoresis (CE). These limitations include the inability to analyze different types of genetic polymorphisms in a single reaction and workflow, low-resolution genotyping, loss of data in degraded DNA samples, and incomplete resolution of complex DNA mixtures [10].

Massively parallel sequencing (MPS) technology has replaced Sanger sequencing in basic science and in medical and diagnostic fields [11–21]. Advantages of MPS in forensic applications include high-throughput processing, simultaneous detection of STRs on autosomes and sex chromosomes, analysis of identity informative SNPs (iiSNPs) and SNPs related to biogeographical ancestry (aiSNPs) and physical traits (phenotypes; piSNPs), and ability to distinguish between alleles that are identical by CE-based sizing. MPS advantages broaden the possibilities of analyses and may provide critical information in challenging samples for criminal cases, missing person cases, and mass disaster tragedies [22, 23].

This report explores the application of MPS using sequencing by synthesis (SBS) [24] on the MiSeq FGx® sequencer (Verogen, Inc.) to analyze dental remains. The MiSeq FGx sequencer, ForenSeq® DNA Signature Prep Kit, and ForenSeq Universal Analysis Software were developed and forensically validated [25, 26] for human identification and generation of investigative leads [27–29]. The ForenSeq DNA Signature Prep Kit contains two PCR primer set options: (1) DNA Primer Mix A (DPMA) targets Amelogenin, 27 autosomal STRs, 24 Y-STRs, 7 X-STRs, and 94 iSNPs and (2) DNA Primer Mix B (DPMB) targets all loci in DPMA, as well as 56 aiSNPs and 22 piSNPs for biogeographical ancestry and phenotype (hair and eye color) estimation [30–34]. Previous evaluations of MiSeq FGx System’s performance and genotyping concordance have been reported [35–41].

Majority of the amplicons in the ForenSeq kit are shorter in base pair (bp) length compared to CE assays, providing a significant advantage when analyzing degraded DNA samples. This is possible because MPS is not limited by the spectral overlap of fluorescent dyes as is fragment sizing-based CE detection. Of the 231 markers targeted with ForenSeq DPMB, 191 total STR and SNP markers have *maximum amplicon sizes* less than 200 bp in length [27]. As a comparison, GlobalFiler®, PowerPlex® Fusion 6c, and MiniFiler® have approximately nine, eight, and six *maximum STR amplicon sizes* that are less than 200 bp, respectively [42–44]. A practical example of the utility of ForenSeq’s smaller amplicon sizes, relative to CE-based methods, on degraded (ancient) remains was reported by Xavier and Parson [38].

Due to hard structures such as enamel and bone that provide environmental protection, dental tissues are a valuable source of genomic and/or mitochondrial DNA, in cases when other biological fluid or tissue may not be available [45]. Few studies have reported on the application of MPS technology to nuclear DNA analysis of dental tissues [38, 45, 46]. Here, we report on evaluation of nuclear DNA utility as obtained from human dental tissues assayed with DFK, InnoQuant HY, and the MiSeq FGx Forensic Genomics System for identification purposes and to generate investigative leads.

## Materials and methods

### Sample collection

#### Teeth samples

Dental samples were obtained from a non-random experimental group of living donors selected according to inclusion and exclusion criteria (see below). The research was undertaken according to the ethical guidelines and approval from the Research Ethics and Compliance Committee of The Dental School of the Universidad de Los Andes (Santiago, Chile). Samples were collected under sterile conditions by dental specialist and placed directly into individually labeled sterile tubes (stored at 20–23 °C (room temperature) and 50–55% humidity).

Ten teeth were collected from donors of the Dental Clinic Cedin (Paine city, Chile; Table 1). Volunteer donors (and parent of the donor if a minor) signed informed consent and/or an assent specifically written for this research. All samples were anonymized. Inclusion criteria were teeth with extraction indication for orthodontics and for dental rehabilitation from donors of both genders, and from ages ranging from 10 to 60 years old. Post mortem intervals (PMI) of extracted teeth were considered between 7 days and 6 months. Exclusion criteria were teeth with caries, clogged, and/or fractured.

**Table 1:** Characteristics for teeth samples (*n* = 10)

Sample code	PMI^a^	Donor age (years)	Gender	Tooth number^b^	Tooth type^c^
3B	5 M, 28 D	12	Male	3.7	SM
4	1 M, 17 D	17	Male	1.4	PM
11	1 M, 12 D	30	Female	4.4	PM
7A	1 M, 4 D	14	Male	1.4	PM
7B	1 M, 4 D	14	Male	4.4	PM
25A	27 D	51	Male	2.4	PM
13	23 D	15	Female	2.4	PM
20	22 D	19	Male	3.8	TM
30	8 D	46	Female	1.1	CI
29	7 D	39	Female	3.8	TM

^a^*PMI*, post mortem interval of tooth; *M*, months and *D*, days

^b^*Tooth number*, FDI nomenclature

^c^Tooth type: *CI*, central incisor; *PM*, premolar; *SM*, second molar; *TM*, third molar

#### Blood samples

Anonymous blood samples from a single male donor were obtained from The Blood Center (New Orleans, LA) for additional testing of moderately to severely degraded DNA.

### Dental tissue sample recovery with the DFK Kit

Dental samples were extracted with the DFK Kit under the same conditions previously described in the “Material and methods” section reported by Carrasco *et al.* [1, 2] (US 61/826, 558 23.05.2013) [3]. Specimen images were captured using digital radiography. The teeth were then externally rehydrated, followed by slight perforation and internal rehydration of the dentin-pulp complex. The rehydrated dental pulp (P) soft tissue was removed from each tooth using a low-speed rotary tool with individual endodontic files (1.5/25 mm SAF, Redent Nova™). External rehydrated root cement (CR) tissue was retrieved (providing a second sample for each tooth) in slices from the radicular surface of the tooth using a no. 15 sterile scalpel. Dental tissues were deposited on a petri dish and subsequently washed with sterile DNase-free water. Each step, including trepanation, tissue recovery, and washing, was conducted in a laminar flow hood under negative pressure to minimize the sample contamination potential. One root cement (CR) and one dental pulp (P) tissue sample was prepared from each of the 10 teeth listed in Table 1, yielding a total of 20 distinct tissue samples. Sample 13 P was consumed during histological analysis for PMI estimation (data not shown) and so was not processed for DNA, leaving 19 distinct tissue samples for DNA analyses.

### DNA extraction

#### Teeth samples

Genomic DNA was extracted from the 19 dental tissue samples (CR and P tissues) using the one-step, 1-hour QuickExtract FFPE DNA Extraction Kit (Lucigen) according to the manufacturer’s instructions [6]. Liquid handling was performed using disposable, sterile filtered tips, and sterile tubes (Axygen, Union City, CA, USA). All operators wore nitrile gloves and disposable masks and caps. A DNA extraction negative control (reagent blank) was included during DNA extraction.

#### Blood samples

One anonymous donor blood sample was extracted using organic methods (Protein Kinase (ProK) and SDS digestion with phenol-chloroform extraction) and purified by cold ethanol precipitation followed by resuspension in Tris-EDTA buffer (TE^−4^ 10 mM Tris-0.1 mM EDTA, pH 8.0). Ten progressively degraded blood samples were created by subjecting the extracted DNA to sonication at 50 °C for time spans ranging from zero to 16 h. Initial assessment of the artificially degraded blood samples enabled evaluation of workflow capabilities prior to application on real-life teeth samples in their natural state.

### DNA quantification

#### Teeth samples and blood samples

Total genomic DNA (gDNA) from the 19 teeth extracts was quantified (once per sample) with the Nanodrop™ 2000 UV-Vis spectrophotometer (Thermo Fisher Scientific™) [47]. The Nanodrop quant values (Table 2) were used to inform CE-based testing (the “Genotyping of STRs and SNPs” section).

**Table 2:** NanoDrop quantification values for 20 collected teeth samples (CR and P tissues)

Sample code	PMI^a^	CR gDNA (ng/μL)^b,c^	P gDNA (ng/μL)^d^
3B	5 M, 28 D	538.5	29.3
4	1 M, 17 D	63.9	487.1
11	1 M, 12 D	83.4	73
7A	1 M, 4 D	65.6	2988.1
7B	1 M, 4 D	365.8	59.3
25A	27 D	37.7	68
13	23 D	59	N/A
20	22 D	116.2	93.4
30	8 D	51.9	157.7
29	7 D	545.2	45.8

^a^*PMI*, post mortem interval of tooth; *M*, months and *D*, days

^b^*CR*, root cement

^c^*gDNA*, genomic DNA

^d^*P*, dental pulp

*N/A*, not available; sample used for histological analysis of PMI (data not shown)

Sufficient volume from thirteen of the original 19 teeth extracts remained after CE-testing. These 13 teeth extracts (Table 3) as well as the 10 progressively degraded blood samples (Table 4) were quantified (once per sample) with InnoQuant HY real-time qPCR (InnoGenomics Technologies) according to manufacturer’s instructions [9] to assess total human DNA and total male DNA (male:female ratio), degradation state, and the presence of PCR inhibitors. InnoQuant HY short and long target concentrations (ng/μL) and the corresponding degradation index (DI = [Short/Long]) for each sample were used to inform MPS-based testing (the “Genotyping of STRs and SNPs” section).

**Table 3:** InnoQuant HY long and short target quantification values and degradation indexes (DI) for 13 of the teeth samples (CR and P tissues)

Sample code	PMI^a^	Short target (ng/μL)	Long target (ng/μL)	Degradation index (DI)	Total long target DNA input (ng)
3B CR	5 M, 28 D	865.3	724.9	1.2	3.6
4 CR	1 M, 17 D	2.6	1.5	1.7	4.6
4 P	1 M, 17 D	1313.4	1481.7	0.89	1.5
11 CR	1 M, 12 D	21.7	21.4	1.01	2.1
11 P	1 M, 12 D	811.0	1251.0	0.65	2.5
7B CR	1 M, 4 D	0.01	0.0001	75.6	0.001
7A P	1 M, 4 D	413.7	286.7	1.4	5.7
25A CR	27 D	0.31	0.21	1.5	1.1
25A P	27 D	488.1	465.0	1.1	2.3
13 CR	23 D	0.01	0.009	1.5	0.05
20 CR*	22 D	0.008	0.002	3.4	0.01
30 P	8 D	565.2	501.5	1.1	2.5
29 P	7 D	2.1	0.27	7.8	1.4

^a^*PMI*, post mortem interval of tooth; *M*, months; *D*, days

*20 CR, re-quantification values after concentration

*CR*, root cement; *P*, dental pulp

**Table 4:** InnoQuant HY long and short target quantification values and degradation indexes (DI) for 10 artificially and progressively degraded blood samples (AD_1 being the least degraded and AD_10 the most degraded)

Sample code	Short target (ng/μL)	Long target (ng/μL)	Degradation index (DI)	Total long target DNA input (ng)
AD_1	0.87	0.87	1.0	1.0
AD_2	0.78	0.35	2.2	1.7
AD_3	0.65	0.17	3.9	0.84
AD_4	1.9	0.15	12.6	0.77
AD_5	0.28	0.02	15.6	0.09
AD_6	0.42	0.02	26.3	0.08
AD_7	0.37	0.01	36.8	0.05
AD_8	0.33	0.007	48.6	0.03
AD_9	0.17	0.001	160.3	0.006
AD_10	0.17	0.0004	459.8	0.002

### Genotyping of STRs and SNPs

#### CE-based genotyping by sizing—teeth samples

Genotyping was first conducted on the 19 teeth samples using capillary electrophoresis (CE) with the Identifiler® Plus PCR Amplification Kit (Thermo Fisher Scientific) targeting 1 ng total input for 15 STRs plus Amelogenin on a 3100® Genetic Analyzer (Thermo Fisher Scientific) [48, 49]. CE data were analyzed using GeneMapper® IDX v1.4 software (Thermo Fisher Scientific) with default stutter filters and an analytical threshold of 50 RFU [50]. Length-based allele designations were named according to the recommendations of the DNA Commission of the International Society of Forensic Genetics (ISFG) [51].

#### MPS-based genotyping by sequencing—blood and teeth samples

Prior to conducting MPS analysis on the 13 teeth extracts that had not been consumed during CE-testing, a preliminary degradation study was conducted on the progressively degraded blood samples, to verify and optimize the genotyping performance of the MiSeq FGx System when utilizing InnoQuant HY degradation index-informed quantification values for ForenSeq amplification. ForenSeq DNA Primer Mix B (DPMB) sequencing libraries (230 targeted STR and SNP markers plus Amelogenin) were prepared according to manufacturer’s instructions for the 10 artificially degraded blood samples using the ForenSeq DNA Signature Prep Kit [27]. Samples were amplified according to long and short target quantification values (*n* = 20), utilizing a maximum DNA input of 5 μL, followed by sequencing on the MiSeq FGx Instrument [28] at a 32-sample multiplex (20 blood samples, 10 samples not related to this study, and one positive and negative amplification control). Data analysis was conducted using the ForenSeq Universal Analysis Software; default STR stutter filter values were utilized and a single allele calling threshold (default analytical threshold) was employed for all STR and SNP markers [29].

Following this initial degradation assessment of blood samples, MPS genotyping was conducted with the MiSeq FGx System for the 13 teeth samples (CR and P tissues) that were quantified with InnoQuant HY. ForenSeq DPMB libraries were prepared, utilizing a maximum of 5 μL of DNA input for amplification according to InnoQuant HY long target quantification values. A 27-sample multiplex run was performed on the MiSeq FGx Instrument (20 total replicates of the dental samples (includes duplicates), three positive amplification controls and four negative controls) followed by data analysis in the ForenSeq Universal Analysis Software; default STR stutter filter values were utilized and a single allele calling threshold (default analytical threshold) was employed for all STR and SNP markers. A second round of analysis was conducted on one of the 13 teeth extracts (sample 20 CR) due to a zero long target input quantification result and therefore lower typing results compared to the other samples. Sample 20 CR was concentrated utilizing DNA Clean and Concentrator™-25 (Zymo Research, Irvine, CA) followed by re-quantification with InnoQuant HY and reanalysis with the MiSeq FGx System (ForenSeq DPMB amplification followed by an 8-sample multiplex run on the MiSeq FGx Instrument to increase sequencing coverage).

For both samples sets, artificially degraded blood and dental tissue samples, the total number of STR alleles and iSNP loci typed were calculated. Autosomal STR genotype concordance was assessed by comparing ForenSeq autosomal STR allele calls to CE-generated autosomal STR results, as available.

Biogeographical ancestry and phenotype (hair and eye color) estimation was performed using the ForenSeq Universal Analysis Software (based on results obtained from targeting 22 aiSNPs, 56 piSNPs, and 2 common SNPs using the default analytical calling threshold).

Random match probability (RMP) population statistics were calculated using the ForenSeq Universal Analysis Software for autosomal STR and iSNP genotype results. Two blood and teeth sample results with the lowest amount of genotype data detected were calculated (samples AD_10 and 20CR) to describe the discriminatory power of ForenSeq data even when partial profile results are obtained. Length-based population allele frequency data from Novroski et al. and Churchill et al. (default installed within the ForenSeq Universal Analysis Software) were utilized to calculate autosomal STR and iSNP RMP statistics for the Caucasian population dataset [36, 52]. A theta value of 0.01 was utilized and the 2p-p^2^ calculation was performed on all homozygous results due to the lower level and potentially stochastic nature of the data.

## Results and discussion

### Quantification results

#### Blood samples

Ten progressively degraded blood samples quantified with InnoQuant HY resulted in short target concentrations ranging from 0.87 to 0.17 ng/μL and long target concentrations from 0.87 to 0.004 ng/μL (Table 4). The degradation indexes (DI) ranged from one (no degradation indicated) to 460 (severe degradation indicated). ForenSeq amplification according to long target quant values (using a maximum of 5 μL DNA extract input volume) yielded total long target DNA inputs from 2 pg to 1.7 ng.

#### Teeth samples

The 19 teeth samples (10 CR and 9 P tissue samples recovered) were initially quantified with NanoDrop, resulting in concentrations ranging from 29.3 to 2988.1 ng/μL (Table 2). To provide more informative quantification data, 13 of the remaining teeth extracts were subsequently quantified with InnoQuant HY (Table 3). Advantages that were observed of the InnoQuant HY system compared to NanoDrop include the following: human specific results, total human and total male concentrations provided, quality information with degradation indexes, and an IPC for degradation and inhibition detection [8, 9]. InnoQuant HY quantification results indicated that root cement and dental pulp tissues recovered using the DFK method yielded DNA of sufficient quality and quantity for the majority of the teeth samples. Eleven of the 13 samples (85%) had degradation indexes ranging from 0.7 to 3.4, indicating that they were not significantly degraded. The remaining two samples had a DI of 7.8 (sample 29 P) and a DI of 75.6 (sample 7B CR), indicating moderate to pronounced degradation. Long target concentration values were utilized for amplification (with a maximum of 5 μL DNA input volume) and indicated that 10 of the 13 samples (77%) had 1.1 to 5.7 ng of total long target DNA input available for amplification and sequencing (Table 3). The remaining three tooth samples ranged from 1 to 50 pg total long target DNA input.

Amplifying according to the long target value may be advantageous, particularly when degradation is present, to enrich for more total DNA input that is viable for amplification. With CE-based methods, this advantage is limited by the risk of saturating the CCD camera when excessive input DNA is utilized (i.e.*,* off-scale data, spectral bleed-through). With ForenSeq processing, higher PCR template amounts (i.e.*,* greater than the recommended 1 ng total input) may be amplified, in an attempt to increase allele detection in degraded samples. Long target quant values were utilized for the ForenSeq data presented herein (with a maximum of 5 μL DNA extract input volume to amplification).

### CE (STR) and NGS (STR and iSNP) genotyping results

#### Blood samples

**Fig. 1:**
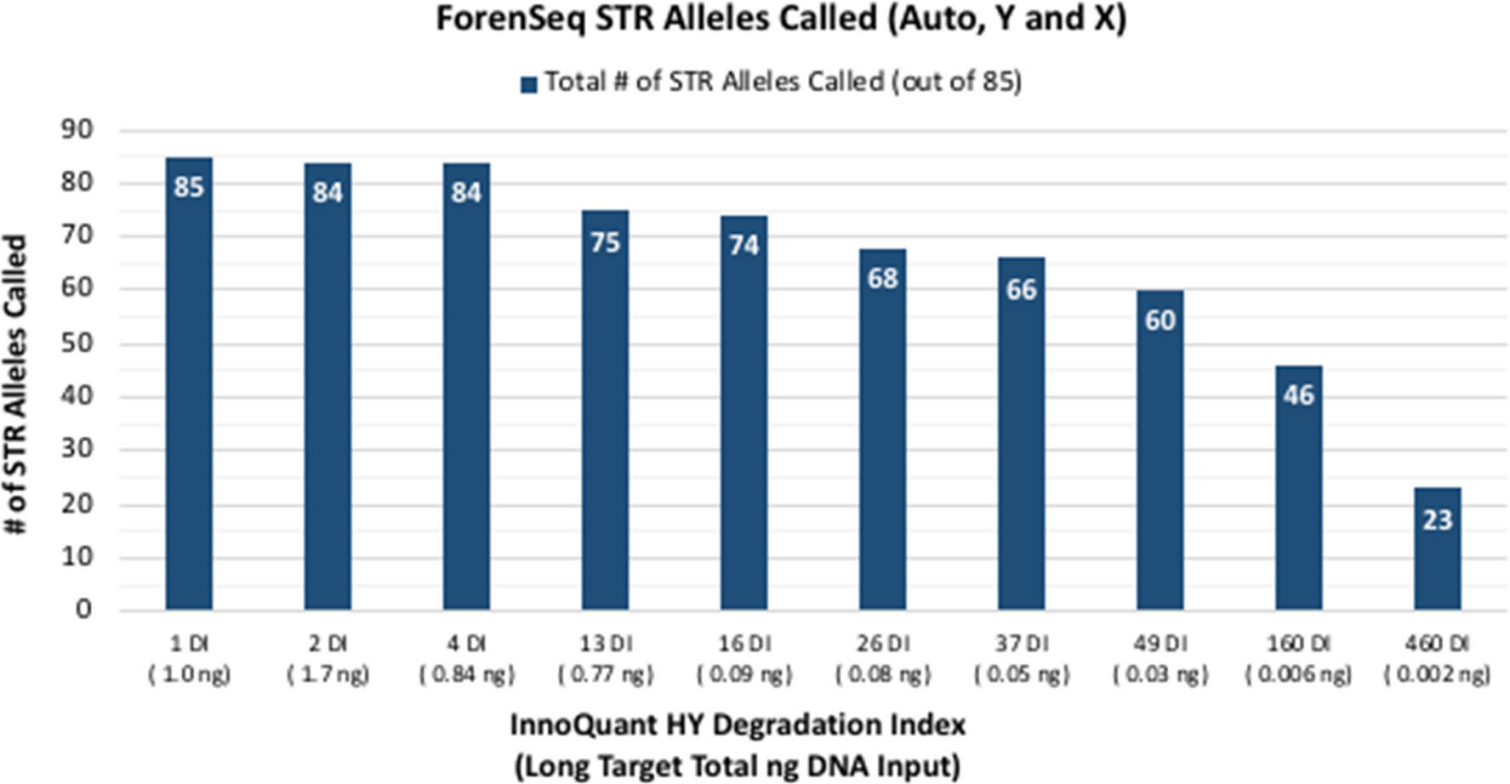
Total number of STR alleles (Autosomal, Y, and X) detected for artificially degraded blood samples with DI ranging from 1 (not degraded) to 460 (severely degraded) and total long target DNA inputs ranging from 1.7 ng to 2 pg

**Fig. 2:**
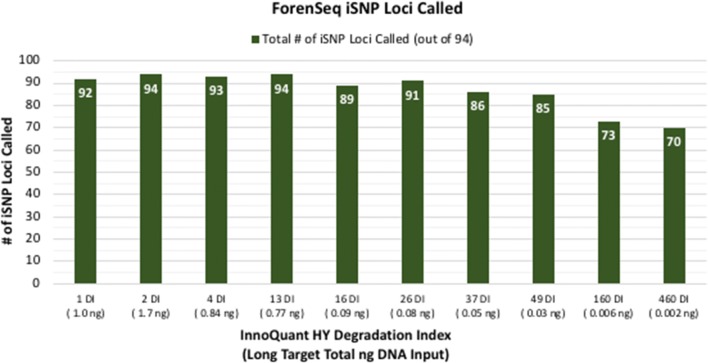
Total number of iSNP loci called for artificially degraded blood samples with DI ranging from 1 (not degraded) to 460 (severely degraded) and total long target DNA inputs ranging from 1.7 ng to 2 pg

Figures 1 and 2 show the total number of STR alleles called and iSNP loci typed for 10 progressively degraded blood samples processed with the MiSeq FGx System (DPMB ForenSeq libraries on a 32-sample multiplex run). The total number of autosomal, Y- and X-STR alleles called for each sample ranged from 85 (100%) with a DI of one at one ng total long target DNA input to 23 alleles (27%) with a 460 DI and two pg total long target DNA input (Fig. 1). Total iSNP loci typed ranged from 70 to 94 (74–100%) (Fig. 2).

#### Teeth samples

**Fig. 3:**
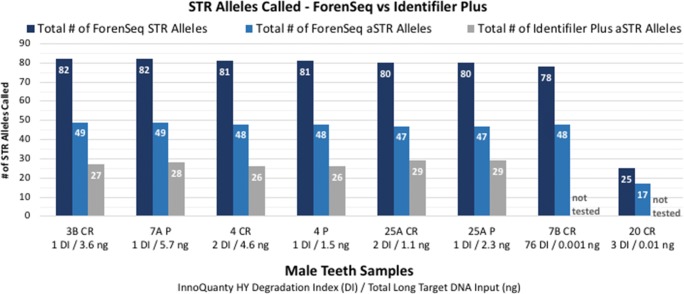
Total number of STR allele calls for ForenSeq vs Identifiler Plus in male teeth samples (*n* = 8). ForenSeq targets 27 autosomal STR, 24 Y-STR, and 7 X-STR loci plus Amelogenin. Identifiler Plus targets 15 autosomal STR loci plus Amelogenin

**Fig. 4:**
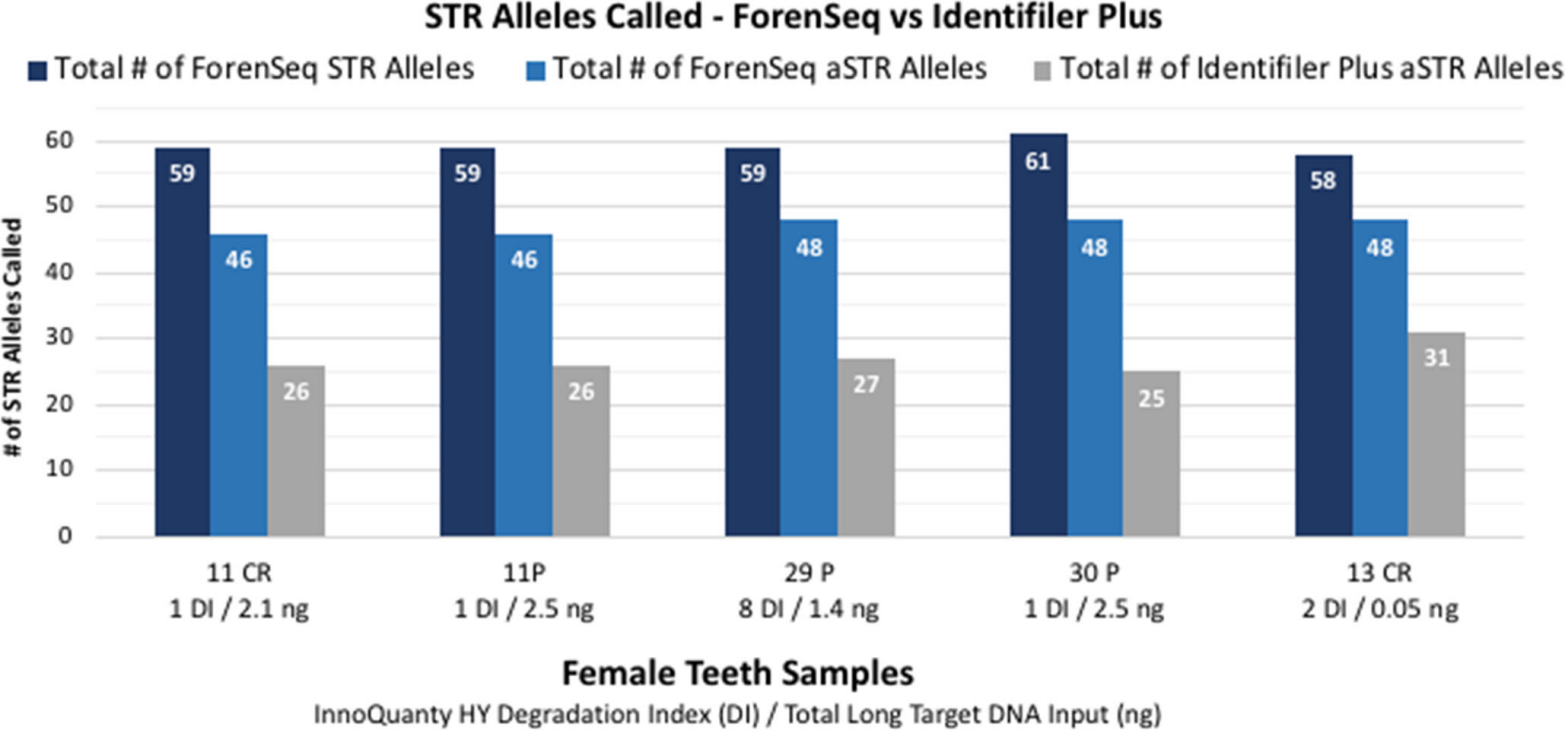
Total number of STR allele calls for ForenSeq vs Identifiler Plus in female teeth samples (*n* = 5). ForenSeq targets 27 autosomal STR, 24 Y-STR, and 7 X-STR loci plus Amelogenin. Identifiler Plus targets 15 autosomal STR loci plus Amelogenin

**Fig. 5:**
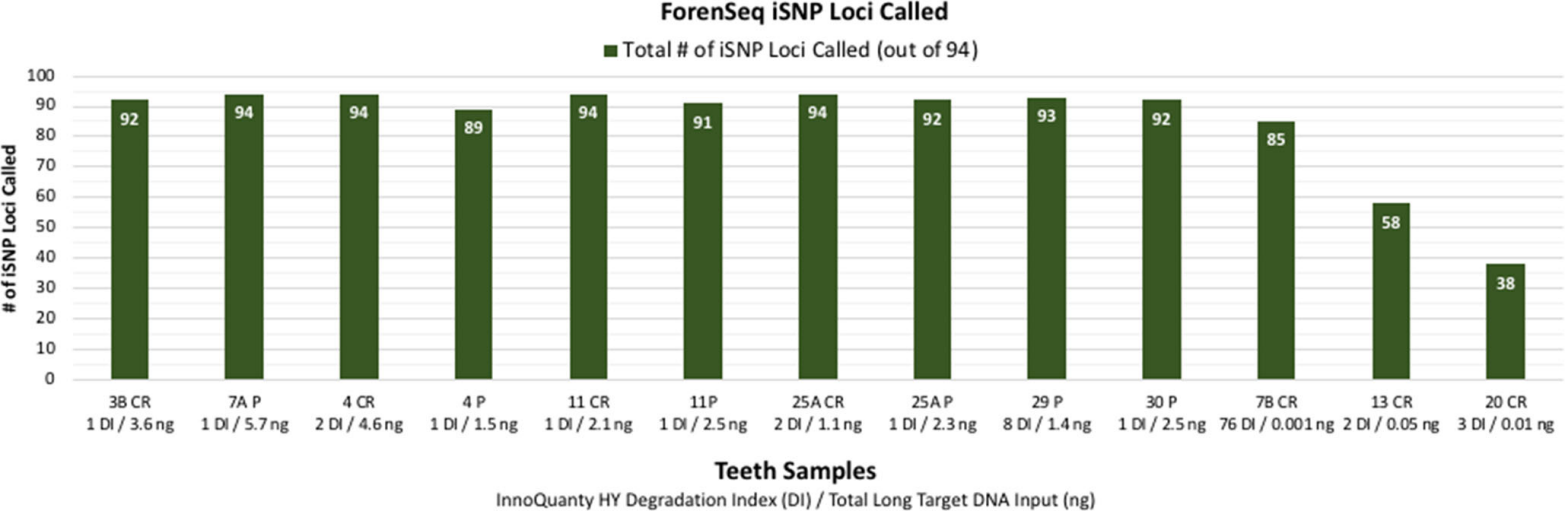
Total number of iSNP loci called with ForenSeq for teeth samples (*n* = 13)

Eleven of the 13 teeth samples were typed with both Identifiler Plus and ForenSeq DNA Signature (DPMB, 27-sample multiplex on MiSeq FGx) and resulted in concordant profiles when the 16 overlapping (redundant) autosomal STR loci plus Amelogenin were compared. Of the 176 loci compared, two observed differences were attributed to allele drop-out at one ForenSeq locus (D5S818 in sample 13 CR) and allele drop-out at one Identifiler Plus locus (D19S433 in sample 7A P). Figures 3 and 4 indicate total number of STR allele calls for both Identifiler Plus (targets 15 autosomal STR loci plus Amelogenin) and ForenSeq (targets 27 autosomal STR, 24 Y-STR, and 7 X-STR loci plus Amelogenin). Ten of the 13 teeth extracts were not significantly degraded (DI ranged from one to eight) and contained at least 1 ng total long target DNA input, resulting in: 46 to 49 ForenSeq autosomal STR allele calls (for 27 total autosomal STR loci targeted plus Amelogenin), compared to 25 to 29 autosomal STR allele calls with Identifiler Plus (15 autosomal STR loci targeted plus Amelogenin). ForenSeq amplifies seven X-STR and 24 Y-STR loci in addition to autosomal STRs, resulting in 80 to 82 total STR allele calls in the non-degraded/higher input male teeth samples and 59 to 61 total STR allele calls in the female samples (no Y-STRs present). Identity SNP loci are simultaneously typed with STRs in the ForenSeq kit (Fig. 5) and resulted in 89 to 94 iSNP loci typed (out of 94 total targets) for the 10 higher quantity/quality teeth samples (DI of one to eight with at least 1 ng total long target DNA input).

Three of the more challenging teeth samples consisted of 7B CR and 20 CR (processed with ForenSeq only) and 13 CR (processed with ForenSeq and Identifiler Plus). Sample 7B CR (DI of 76 and total long target DNA input of 0.001 ng) resulted in 78 total ForenSeq STR allele calls (48 autosomal STR, 24 Y-STR, 6 X-STR alleles) and 85 iSNP loci typed (out of 94 iSNP loci targeted). Sample 20 CR initially resulted in a zero long target concentration (ng/μL) result with InnoQuant HY. The first round of MiSeq FGx processing (27-sample multiplex MiSeq FGx run) resulted in a total of two STRs and zero iSNPs typed, as expected with the negative quantification result. Concentration (via Zymo DNA Clean and Concentrator-25) of sample 20 CR and re-quantification yielded a DI of 3 with long target concentration of 0.002 ng/μL (0.01 ng total long target DNA input to ForenSeq amplification). Sequencing with an 8-sample multiplex, to improve coverage with a known low-level sample, resulted in 25 total ForenSeq STR allele calls (17 autosomal STR, 5 Y-STR, 3 X-STR alleles) and 38 iSNP loci called (out of 94 iSNP loci targeted). Sample 13 CR (DI of 2 and total long target DNA input of 0.05 ng) resulted in 58 total ForenSeq STR allele calls (48 autosomal STR, 10 X-STR alleles; female sample) and 58 iSNP loci typed (out of 94 iSNP loci targeted). Comparatively, Identifiler Plus generated 31 autosomal STR allele calls.

### Population frequency statistics

Autosomal STR and iSNP random match probability (RMP) population statistics (Caucasian) generated by the ForenSeq Universal Analysis Software for blood sample AD_10 (12 autosomal STR and 70 iSNP loci typed) and tooth sample 20CR (13 autosomal STR and 38 iSNP loci typed) are provided in Supplemental Figs. 1 and 2, respectively. Blood sample AD_10 resulted in an iSNP RMP of 1 in ~ 2.9 trillion, and an autosomal STR RMP of 1 in ~ 202 million. Tooth sample 20 CR resulted in an iSNP RMP of 1 in ~ 350,000, and an autosomal STR RMP of 1 in ~ 860 million. These results demonstrate the practical casework utility and discriminatory power of ForenSeq profiles when minimal, partial profile data are obtained from challenging samples. It should be noted that these results utilize length-based allele frequency data; further discriminatory power is achieved when the sequence-based population allele frequency data are employed [53].

### Biogeographical ancestry and phenotype estimation

In addition to STR and iSNP data for identity testing, ForenSeq DPMB sequencing results include biogeographical ancestry-informative and phenotypic-informative SNPs (aiSNPs and piSNPs, respectively) for the generation of investigative genetic leads. These data provide biogeographical ancestry and phenotype (hair and eye color) estimation in the ForenSeq Universal Analysis Software.

#### Blood samples

For the 10 artificially degraded blood samples: hair and eye color estimations, which require 100% piSNP locus call rates (22 piSNPs and 2 common SNPs targeted), were generated for the first five samples in the degradation series down to 90 pg total long target DNA input with a DI of 16. Hair color estimations for these five samples originating from the same anonymous blood donor all indicated 74% red, 22% blond, 4% brown, and 0% black. Eye color estimations were in the range of 95–97% blue, 2–3% intermediate, and 1–2% brown. Biogeographical ancestry estimation was consistently depicted as the European population group (see Supplemental Fig. 3 for an example) for all 10 samples, with 100% locus call rates obtained for seven of the 10 samples and 80–96% locus call rates for the remaining three samples (54 aiSNPs and 2 common SNPs targeted). A minimum of one aiSNP allele call is required for a biogeographical ancestry estimation to be generated; however, decreasing call rates may result in diminished accuracy of the estimation. aiSNP and piSNP locus call rate data and estimation results for all 10 blood samples are detailed in Supplemental Table 1.

#### Teeth samples

**Fig. 6:**
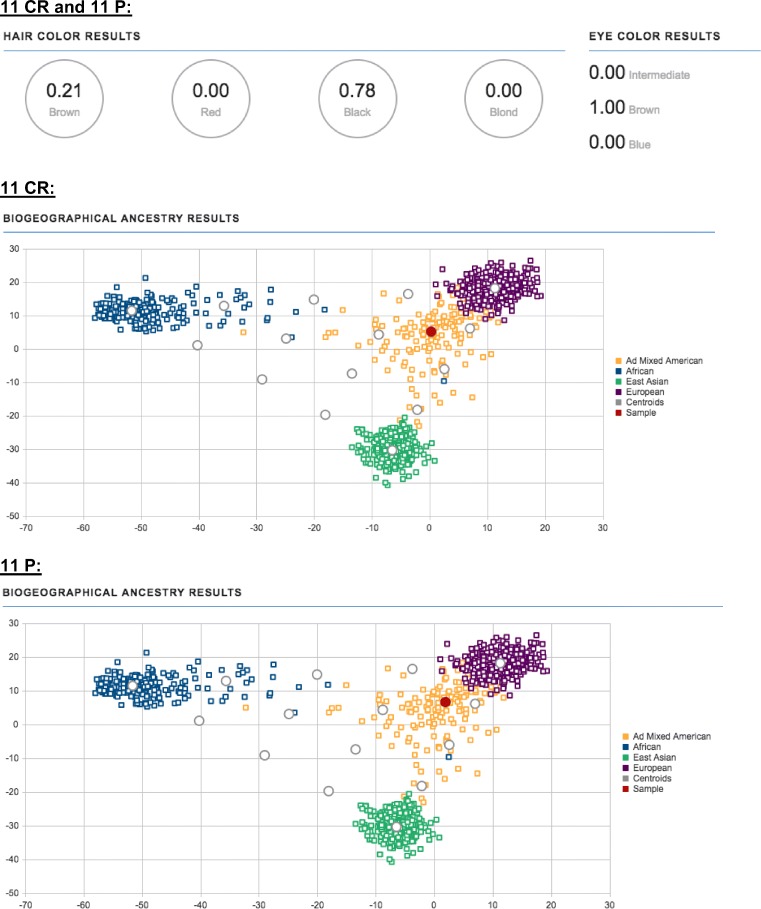
Visible trait (hair and eye color phenotypes) and biogeographical ancestry estimations in the ForenSeq Universal Analysis Software: teeth samples 11 CR and 11 P (root cement and dental pulp extracted from the same tooth) (samples indicated with a red dot)

For the 13 teeth samples (root cement and dental pulp tissues), piSNP locus call rates ranged from 46–100%, with five of the samples having 100% piSNP locus call rates available for hair and eye color estimations (Supplemental Table 2). aiSNP locus call rates ranged from 39–100%, with all 13 samples resulting in an AdMixed American population result. Figure 6 demonstrates consistent conclusions for phenotype and biogeographical ancestry estimations for teeth samples 11 CR and 11 P (root cement and dental pulp extracted from the same tooth), and the results are as expected based on the known/observed phenotype and biogeographical ancestry of the donor recorded during sample collection (dark brown/black hair, brown eyes, and South American descent).

## Conclusions and future directions

The data described in this study demonstrate the robust performance of a comprehensive sample-to-sequencing workflow capable of achieving informative nuclear DNA results from challenging, low-level, and/or degraded samples. The DFK method provided efficient recovery of the dental pulp and root cement tissues from teeth samples, resulting in two samples per tooth suitable for nuclear DNA extraction (and other types of forensic examination) while preserving the tooth. InnoQuant HY quantification of QuickExtract FFPE Kit extracted teeth DNA and organic extracted blood samples provided a highly sensitive and accurate assessment of nuclear DNA quantity and quality; actionable results were obtained for teeth samples down to 0.01 ng/μL short target and 0.0001 ng/μL long target concentrations with corresponding DI of 76, and for blood samples down to 0.17 ng/μL short target and 0.0004 ng/μL long target concentrations with corresponding DI of 460. The accurate quant-based characterization of DNA quality and quantity enabled data-informed amplification targeting 231 STR and SNP markers with the ForenSeq DNA Signature Prep Kit.

Compared with CE amplification kits, ForenSeq provides a larger multiplex with smaller amplicon sizes (191 STR and SNP makers with maximum amplicon size ≤ 200 bp) to more effectively type degraded DNA samples. Sequencing of 13 teeth samples on the MiSeq FGx instrument generated 24 to 82 total STR allele calls per sample (58 STR loci targeted plus Amelogenin) and simultaneously typed 38 to 94 iSNP loci per sample (94 iSNP loci targeted). The tooth sample that yielded the least amount of data (22% of autosomal STR and 40% of iSNP loci typed) resulted in an autosomal STR RMP of 1 in ~ 860 million and an iSNP RMP of 1 in ~ 350,000. Sequencing of the most severely degraded blood sample (DI of 460 with 2 pg total long target DNA input) generated data for 21% of autosomal STR loci and 74% of iSNP loci, with a corresponding autosomal STR RMP of 1 in ~ 202 million and iSNP RMP of 1 in ~ 2.9 trillion. These results demonstrate highly discriminatory genotype frequency statistics even with diminishing data recovery in challenging samples.

Collectively, application of the DFK method for tissue recovery and DNA extraction, followed by InnoQuant HY quantification and MiSeq FGx System genotyping of dental samples generates a significant amount of usable data for successful sample identification, with the added benefit of potential investigative leads through biogeographical ancestry and phenotype estimation. The demonstrated success of this three-part comprehensive workflow on teeth samples and degraded DNA suggests that the application of these combined methods may also benefit nuclear DNA processing results for aged skeletal remains (i.e., bone samples). The DFK, InnoQuant HY, and MiSeq FGx workflows were employed in a preliminary test of one 45-year PMI jawbone sample resulting in the detection of 48 STR alleles and 59 iSNP loci. Further evaluation to verify the success of this method with bone samples is warranted. Additionally, comparison of different extraction methods (e.g. organic vs QuickExtract FFPE) utilized on the DFK recovered tissues, as well as the addition of a purification/concentration step (e.g.*,* filter concentrators) may further optimize the workflow and improve results for the most challenging scenarios.

## Electronic supplementary material

ESM 1 (DOCX 3.01 mb)
