# Supplementary material for: Optimizing DNA recovery and forensic typing of degraded blood and dental remains using a specialized extraction method, comprehensive qPCR sample characterization, and massively parallel sequencing
Source: Int J Legal Med. 2019 Aug 14;134(1):79–91. doi: 10.1007/s00414-019-02124-y (PMC6949324; doi:10.1007/s00414-019-02124-y)
Supplement: Supplementary file 1 — (DOCX 3.01 mb) [file 414_2019_2124_MOESM1_ESM.docx]

**Supplemental Figure 1:** ForenSeq Universal Analysis Software iSNP (top) and autosomal STR (bottom) RMP population statistics for blood sample AD_10 (460 DI with 2 pg total long target DNA input); 12 autosomal STR loci and 70 iSNP loci typed)


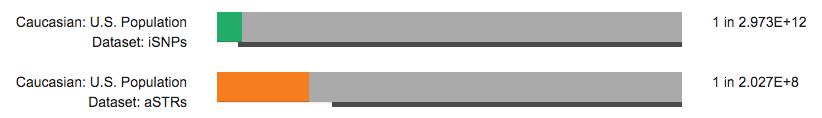


**Lower dark gray bar** – user-defined source attribution threshold indication

**Green result bar** – statistical result is ≥ the user defined source attribution threshold

**Orange result bar** – statistical results is < the user defined source attribution threshold

**Supplemental Figure 2:** ForenSeq Universal Analysis Software iSNP (top) and autosomal STR (bottom) RMP population statistics for tooth sample 20CR (required additional purification/filter concentration); 3 DI with 10 pg total long target DNA input; 13 autosomal STR loci and 38 iSNP loci typed.


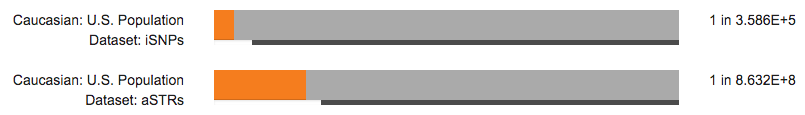


**Lower dark gray bar** – user-defined source attribution threshold indication

**Orange result bar** – statistical results is < the user defined source attribution threshold

**Supplemental Figure 3:** Visible trait (hair and eye color phenotypes) and biogeographical ancestry estimations in the ForenSeq Universal Analysis Software: degraded blood sample, 90 pg total long target DNA input, and a DI of 16 (sample indicated with red dot).


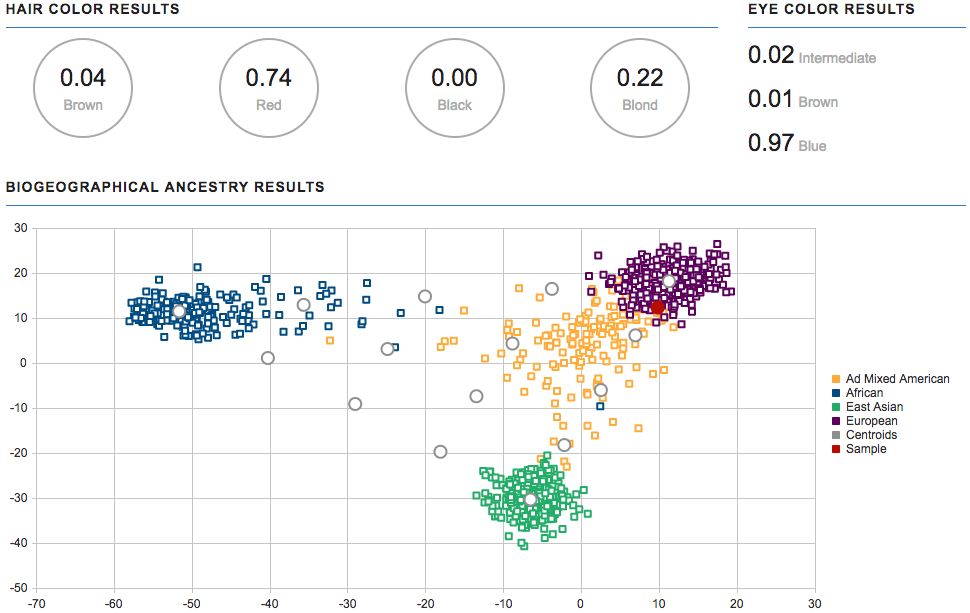


**Hair Color Results** – Hair phenotype percentages based in the HIrisPlex model described by Walsh et al. [30]

**Eye Color Results** – Eye color phenotype percentages based in the HIrisPlex model described by Walsh et al. [30]

**Biogeographical Ancestry Results** – Graph of the result from the principal component analysis of the sample data set. Biogeographical ancestry estimation is obtained by principal component analysis (PCA). The model was trained on the European, East Asian, and African (excepting ASW) super populations of the 1000 Genomes data from Phase I of the project. The unknown sample is projected along with the Ad-Mixed Americans super population, for context, onto the pre-trained first two components based on its aiSNP genotype calls. [33]

**Supplemental Table 1:** aiSNP and piSNP locus call rates (%) and phenotype estimation from ForenSeq Universal Analysis Software for 10 artificially degraded blood samples (same donor).

| Sample Code | DI | Total Long Target DNA Input (ng) | piSNP Locus Call Rate | Hair Color Estimation | Eye Color Estimation | aiSNP Locus Call Rate | Biogeographical Ancestry Estimation |
| --- | --- | --- | --- | --- | --- | --- | --- |
| AD_1 | 1.0 | 1.0 | 100% | 74% Red  22% Blond  4% Brown  0% Black | 95% Blue  3% Int  2% Brown | 100% | European |
| AD_2 | 2.2 | 1.7 | 100% | 74% Red  22% Blond  4% Brown  0% Black | 96% Blue  3% Int  1% Brown | 100% | European |
| AD_3 | 3.9 | 0.84 | 100% | 74% Red  22% Blond  4% Brown  0% Black | 96% Blue  3% Int  1% Brown | 100% | European |
| AD_4 | 12.6 | 0.77 | 100% | 74% Red  22% Blond  4% Brown  0% Black | 96% Blue  3% Int  1% Brown | 100% | European |
| AD_5 | 15.6 | 0.09 | 100% | 74% Red  22% Blond  4% Brown  0% Black | 97% Blue  2% Int  1% Brown | 100% | European |
| AD_6 | 26.3 | 0.08 | 83% | N/A | N/A | 100% | European |
| AD_7 | 36.8 | 0.05 | 83% | N/A | N/A | 96% | European |
| AD_8 | 48.6 | 0.03 | 75% | N/A | N/A | 100% | European |
| AD_9 | 160.3 | 0.006 | 75% | N/A | N/A | 86% | European |
| AD_10 | 459.8 | 0.002 | 46% | N/A | N/A | 80% | European |

**DI:** Degradation Index

**Int:** Intermediate

**N/A:** Not available (piSNPs <100%)

**Supplemental Table 2:** aiSNP and piSNP locus call rates (%) and phenotype estimation from ForenSeq Universal Analysis Software for 13 root cement and dental pulp teeth samples.

| Sample Code | DI | Total Long Target DNA Input (ng) | piSNP Locus Call Rate | Hair Color Estimation | Eye Color Estimation | aiSNP Locus Call Rate | Biogeographical Ancestry Estimation |
| --- | --- | --- | --- | --- | --- | --- | --- |
| 3B CR | 1.2 | 3.6 | 96% | N/A | N/A | 98% | Ad Mixed American |
| 4 CR | 1.7 | 4.6 | 100% | 81% Black  18% Brown  1% Blond  0% Red | 100% Brown  0% Int  0% Blue | 100% | Ad Mixed American |
| 4 P | 0.89 | 1.5 | 92% | N/A | N/A | 91% | Ad Mixed American |
| 11 CR | 1.01 | 2.1 | 100% | 78% Black  21% Brown  0% Blond  0% Red | 100% Brown  0% Int  0% Blue | 100% | Ad Mixed American |
| 11 P | 0.65 | 2.5 | 100% | 78% Black  21% Brown  0% Blond  0% Red | 100% Brown  0% Int  0% Blue | 100% | Ad Mixed American |
| 7B CR | 75.6 | 0.001 | 83% | N/A | N/A | 96% | Ad Mixed American |
| 7A P | 1.4 | 5.7 | 100% | 65% Black  33% Brown  2% Blond  0% Red | 99% Brown  1% Int  0% Blue | 100% | Ad Mixed American |
| 25A CR | 1.5 | 1.1 | 100% | 46% Brown  42% Black  12% Blond  0% Red | 99% Brown  1% Int  0% Blue | 100% | Ad Mixed American |
| 25A P | 1.1 | 2.3 | 96% | N/A | N/A | 96% | Ad Mixed American |
| 13 CR | 1.5 | 0.05 | 67% | N/A | N/A | 61% | Ad Mixed American |
| 20 CR | 3.4 | 0.01 | 46% | N/A | N/A | 39% | Ad Mixed American |
| 30 P | 1.1 | 2.5 | 96% | N/A | N/A | 98% | Ad Mixed American |
| 29 P | 7.8 | 1.4 | 86% | N/A | N/A | 98% | Ad Mixed American |

**DI:** Degradation Index

**Int:** Intermediate

**N/A:** Not available (piSNPs <100%)
